# Additional Evidence That the Polymerase Subunits Contribute to the Viral Replication and the Virulence of H5N1 Avian Influenza Virus Isolates in Mice

**DOI:** 10.1371/journal.pone.0124422

**Published:** 2015-05-04

**Authors:** Xiao Qu, Longfei Ding, Zhenqiao Qin, Jianguo Wu, Zishu Pan

**Affiliations:** State Key Laboratory of Virology, College of Life Sciences, Wuhan University, Wuhan 430072, China; University of North Carolina at Greensboro, UNITED STATES

## Abstract

Genetically similar H5N1 viruses circulating in the avian reservoir exhibit different levels of pathogenicity in mice. In this study, we characterized two highly pathogenic H5N1 avian isolates—A/Hunan/316/2005 (HN05), which is highly pathogenic in mice, and A/Hubei/489/2004 (HB04), which is nonpathogenic. In mammalian cells, HN05 replicates more efficiently than HB04, although both viruses have similar growth kinetics in avian cells. We used reverse genetics to generate recombinant H5N1 strains containing genes from HN05 and HB04 and examined their virulence. HN05 genes encoding the polymerase complex determine pathogenicity and viral replication ability both *in vitro* and *in vivo*. The PB2 subunit plays an important role in enhancing viral replication, and the PB1 and PA subunits contribute mainly to pathogenicity in mice. These results can be used to elucidate host-range expansion and the molecular basis of the high virulence of H5N1 viruses in mammalian species.

## Introduction

The highly pathogenic H5N1 avian influenza viruses (AIVs) circulating in animal reservoirs represent a significant public health threat. H5N1 AIVs have spread over large parts of Asia, Africa and Europe and are occasionally transmitted to mammalian species [1–7]. In the 1997 Hong Kong H5N1 outbreak, viruses isolated from clinical cases could replicate in mice without adaptation, and their virulence in mice was varied [8,9]. Although they are not efficiently transmitted among humans, H5N1 viruses can undergo point mutation or gene reassortment to facilitate airborne transmission among ferrets [10,11]. Therefore, understanding the mechanisms by which influenza viruses acquire the ability to infect multiple species is imperative for controlling future outbreaks.

The pathogenicity of influenza viruses is a polygenic trait that includes contributions from genes encoding hemagglutinin (HA), nonstructural protein (NS) and the polymerase complex [12–17]. A major determinant of viral tropism is the influenza virus polymerase [18]. The polymerase is composed of the viral polymerase subunits PB1, PB2, and PA and assembles with viral RNA and nucleoprotein (NP) to mediate the transcription and replication of the viral genome. The influenza virus polymerase complex is involved in viral virulence and interspecies transmission [19–23]. Viral polymerase subunits from human isolates might not be fully compatible with those isolated from avian strains [24,25], and specific amino acid residues in the polymerase subunits might control host restriction [13,24,26,27].

Mutations in polymerase subunits are the main driving force for AIVs to infect mammals. The PB2 subunit of the viral polymerase is an important host range determinant. A lysine at position 627 of PB2 (627K) correlates with enhanced polymerase activity, viral replication and pathogenicity in mammals [12,27–29]. However, this observation is not absolute because H5N1 viruses with the PB2 627E variant can successfully infect mice and ferrets [8,14,30,31]. Other residues within the PB2 protein, such as 701N, 714R, 158G and 271A, are also important for mammalian host specificity and pathogenicity [21,32–36]. By contrast, very few host adaptive substitutions have been observed in the PB1 and PA subunits. The mutations PB1 L472V and PB1 L598P can partially compensate for the lack of PB2 627K [37]. In some H5N1 isolates, PA has a more dominant effect on viral polymerase activity than PB2 [38]. PA is also associated with the high virulence of H5N1 isolates in mice [39]. Therefore, the contribution of the polymerase complex genes to viral replication and virulence in mammals varies among different H5N1 strains.

H5N1 isolates are divided into nonpathogenic and pathogenic groups in mice; over time, these viruses circulating in ducks acquired the ability to replicate in mice and cause systemic infection and death without adaptation [40]. Genetic reassortment analyses have revealed that the PB2, PA, NA, and NS genes of H5N1 isolates contribute to virulence in mice [22,41]. In this study, we characterized two H5N1 poultry isolates and tested their virulence in chicken and mice. Using reverse genetics, recombinant viruses were generated to examine the molecular basis of viral replication and pathogenicity in mice.

## Materials and Methods

### Cells and viruses

MDCK (Madin-Darby Canine Kidney), 293T (human kidney), DF-1 (chicken embryo fibroblast) and A549 (human lung epithelial) cell lines were obtained from China Central Type Culture Collection (CCTCC, Wuhan, China) and cultured in Dulbecco’s modified Eagle’s medium (DMEM) (Gibco, Grand Island, NY, USA) supplemented with 10% fetal bovine serum (FBS) (Gibco), 100 U/ml penicillin and 0.1 mg/ml streptomycin. All cell lines were incubated at 37°C in 5% CO_2_. The two wild-type (wt) avian influenza viruses used were A/chicken/Hubei/489/2004 (H5N1) (HB04) isolated from a sick chicken and A/duck/Hunan/316/2005 (H5N1) (HN05) isolated from an apparently healthy wild duck. Viral stocks of the H5N1 viruses were propagated in 10-day-old specific-pathogen-free (SPF) embryonated chicken eggs for 24 h at 37°C; the allantoic fluid was harvested, centrifuged for clarification, and stored at -80°C until they were used. Viral titers were calculated using the Reed-Muench method [42]. All experiments with infectious H5N1 viruses were performed under BSL-3 containment.

### Plasmid construction

We used an eight-plasmid reverse genetics system for virus rescue as described previously [43,44]. Briefly, the viral RNA was extracted from virus-containing allantoic fluid using the RNeasy mini kit (QIAGEN, Valencia, CA, USA), and cDNA fragments of the eight viral genes from either the HB04 or HN05 virus were amplified by reverse transcription-polymerase chain reaction (RT-PCR) using a universal primer set [43,45] (Table 1). The amplicons of the eight viral genes from both H5N1 strains were cloned into the dual-promoter plasmid pHW2000, respectively, to generate the eight-plasmid sets encoding viral genes. All constructs containing full viral genomes were completely sequenced for confirmation. The genomic sequences of both HB04 and HN05 viruses are available in GenBank under the accession numbers AY770077 to AY770084 and KP233701 to KP233708, respectively.

**Table 1:** Primer set used for RT-PCR amplification of the eight vRNAs of influenza A viruses.

Genes	Forward Primers (5′→3′)	Reverse Primers (5′→3′)
PB2	BSM-PB2_F	BSM-PB2_R
	TTCGTCTCAGGG**AGCAAAAGCAGG** **TC**	TCGTCTCGTATT**AGTAGAAACAAGG** **TCGTTT**
PB1	BSM-PB1_F	BSM-PB1_R
	TTCGTCTCAGGG**AGCAAAAGCAGG** **CA**	TCGTCTCGTATT**AGTAGAAACAAGG** **CATTT**
PA	BSM-PA_F	BSM-PA_R
	TTCGTCTCAGGG**AGCAAAAGCAGG** **TAC**	TCGTCTCGTATT**AGTAGAAACAAGG** **TACTT**
HA	BSM-HA_F	BSM-HA_R
	TTCGTCTCAGGG**AGCAAAAGCAGG** **GG**	TCGTCTCGTATT**AGTAGAAACAAGG** **GTGTTTT**
NP	BSM-NP_F	BSM-NP_R
	TTCGTCTCAGGG**AGCAAAAGCAGG** **GTA**	TCGTCTCGTATT**AGTAGAAACAAGG** **GTATTTT**
NA	BSM-NA_F	BSM-NA_R
	TTCGTCTCAGGG**AGCAAAAGCAGG** **AGT**	TCGTCTCGTATT**AGTAGAAACAAGG** **AGTTTTT**
M	BSM-M_F	BSM-M_R
	TTCGTCTCAGGG**AGCAAAAGCAGG** **TAG**	TCGTCTCGTATT**AGTAGAAACAAGG** **TAGTTTT**
NS	BSM-NS_F	BSM-NS_R
	TTCGTCTCAGGG**AGCAAAAGCAGG** **GTG**	TCGTCTCGTATT**AGTAGAAACAAGG** **GTGTTTT**

Note: the sequences complementary to the influenza sequences are shown in bold. The underlined nucleotides at the 3′-end represent the segment-specific sequences. The 5′-end has recognition sequences for the restriction endonuclease *BsmB*I.

### Virus rescue

Virus rescue was performed as previously described [32,45,46]. Briefly, DNA and Lipofectamine 2000 (Invitrogen, Carlsbad, CA, USA) were mixed (2 μl of transfection reagent per μg of DNA) in a total volume of 1 ml of Opti-MEM (Invitrogen), incubated at room temperature for 30 min and added to an 80 to 90% confluent monolayer of 293T and MDCK cells in six-well plates. After incubation at 37°C for 6 h, the transfection mixture was removed from the cells, and 2 ml of Opti-MEM containing 1 μg/ml tosylphenylalanine chloromethyl ketone-treated trypsin (TPCK-trypsin; Worthington Biochemical Corporation) was added. After 72 h, recombinant viruses in the supernatants of transfected cells were plaque-purified on MDCK cells. Virus stocks were prepared in embryonated chicken eggs and stored at -80°C. Viruses were detected using a hemagglutination assay, and full-length amplification of all eight segments was performed as described previously [43,44] followed by sequencing by Sangon Biotech Co., Ltd (Shanghai, China) to confirm viral identity.

### Animal experiments

All animal studies were approved by the Institutional Animal Care and Use Committee at Wuhan University.

To determine the pathogenicity of the two viruses in chickens, the intravenous pathogenicity index (IVPI) was tested according to the recommendation of the OIE [47]. Briefly, groups of 12 6-week-old SPF chickens housed in isolator cages were inoculated intravenously with 0.1 ml of a 1:10 dilution of allantoic fluid containing either HB04 (2×10^6^ TCID_50_) or HN05 (5×10^5^ TCID_50_) virus. Chickens were examined at 24-h intervals for 10 days. At each observation, each chicken was scored as 0 if normal, 1 if sick, 2 if severely sick, or 3 if dead. The IVPI is the mean score per bird per observation over the 10-day period.

For analysis of pathogenicity in mice, groups of 6- to 8-week-old female BALB/c mice (Center for Animal Experiment, Wuhan University) were lightly sedated with isoflurane and inoculated intranasally with 50 μl of 2×10^3^ TCID_50_ of each infectious virus in phosphate-buffered saline (PBS). Three mice from each group were euthanized at 1, 3 and 5 days post-infection (dpi), and their lungs were homogenized with cold sterile PBS. Viral titers were determined by measuring the TCID_50_ in MDCK cells. Cells for TCID_50_ determinations were in serum-free complete DMEM supplemented with 1 μg/ml TPCK-trypsin (Sigma, St. Louis, MO, USA).

The 50% mouse lethal dose (MLD_50_) was determined as described previously [9,48]. Briefly, groups of five mice were inoculated with 10-fold serial dilutions containing 10^1^ to 10^5^ or a dilution of 10^5.5^ TCID_50_ of the virus in a 50-μl volume. Mice were monitored daily for weight loss and mortality up to 14 dpi. All mice showing respiratory distress and more than 25% body weight loss were considered to have reached the experimental endpoint and were humanely euthanized. The MLD_50_ values were calculated by the Reed-Muench method [47] and expressed as the TCID_50_ value.

For pathological analyses, mice were inoculated with 2×10^3^ TCID_50_ of virus, and lungs were collected at 5 dpi and fixed with 4% paraformaldehyde, embedded in paraffin, sectioned at a thickness of 5 μm, stained with hematoxylin and eosin (H&E) and then examined microscopically for histopathological changes. Images were obtained using an Olympus microscope with a 20× objective lens. Lung inflammation severity scores were assigned as described previously [48,49].

### Euthanasia of mice

Euthanasia of mice was performed as described previously [50,51]. All infected mice were killed at humane end-points or at the predetermined end of the experiment. The humane end-points were strictly observed according to the scoring system based on the weight loss and symptom severity scale for influenza infection [50,52]. Animals were scored daily, and each individual mouse with a score less than or equal to 3 points was humanely euthanized. All procedures, including inoculation and euthanasia, were performed under anesthesia to minimize the pain and suffering of infected animals.

### Plaque assay

MDCK cells grown to 90–95% confluence in six-well plates were washed twice with PBS and subsequently inoculated with 10-fold dilutions of influenza virus in Opti-MEM supplemented with 1 μg/ml TPCK-trypsin. After 1 h of incubation at 37°C, unbound virus inoculums were removed, and the cells were rinsed with PBS. The cells were overlaid with 2 ml of DMEM supplemented with 0.8% agarose, 0.2% serum albumin and 1 μg/ml TPCK-trypsin. After incubation at 37°C for 3 days, the cells were fixed using 10% formalin for 1 h and stained with a 0.3% crystal violet solution (Sigma).

### RNP minigenome assays

To compare the activities of viral RNP complexes, dual luciferase reporter assays (Promega, Madison, WI, USA) were performed as described previously [46,53]. Briefly, the reporter plasmid pPolI-NS-Luc contains the firefly luciferase open reading frame (ORF) under the control of the human RNA polymerase I (Pol I) promoter and murine Pol I terminator; the luciferase ORF is flanked by noncoding regions of the NS gene of HB04 virus. The RNP complexes composed of PA, PB1, PB2 and NP proteins bind to the noncoding regions of the NS gene and initiate the transcription of the luciferase containing vRNA-like RNA. The RNP activity was measured as described previously [34,54]. Briefly, 293T cells in 24-well plates were transfected with 0.05 μg of the pPolI-NS-Luc plasmid as well as mixtures of the four bidirectional plasmids pHW-PB2, pHW-PB1, pHW-PA, and pHW-NP (0.1, 0.1, 0.1 and 0.2 μg, respectively) of the HB04 or HN05 virus. The *Renilla* luciferase expression plasmid pRL-TK (0.05 μg) was used as an internal control and firefly luciferase activity was normalized by *Renilla* luciferase activity. At 24 h post-transfection, cell lysates were prepared using the Dual-Luciferase Reporter Assay System (Promega), and luciferase activity was measured using a GloMax 20/20 luminometer. Each luciferase activity value is the average of three independent experiments.

### Replication kinetics *in vitro*

To examine multi-step growth, A549, MDCK and DF-1 cell monolayers in 24-well plates were washed twice with PBS and infected with reassortant viruses at a multiplicity of 0.1, 0.01, or 0.01 PFU/cell, respectively. After a 1-h incubation, the inoculum was removed, and the monolayers were washed with PBS, replenished with 2 ml of serum-free complete DMEM supplemented with TPCK-treated trypsin, and re-fed with the same medium. The final concentrations of TPCK-trypsin were 1.0 μg/ml for MDCK cells and 0.1 μg/ml for A549 or DF-1 cells. The plates were then incubated at 37°C as indicated, and supernatants were collected at 8, 12, 24, 48, and 72 h post-infection (p.i.). Viral titers were determined using MDCK cells.

### Statistical analysis

Statistical analysis of the data was performed using Student’s *t* test. A *p*-value less than 0.05 was considered statistically significant.

## Results

### Pathogenicity of two avian H5N1 isolates in chicken and mice

**Fig 1:**
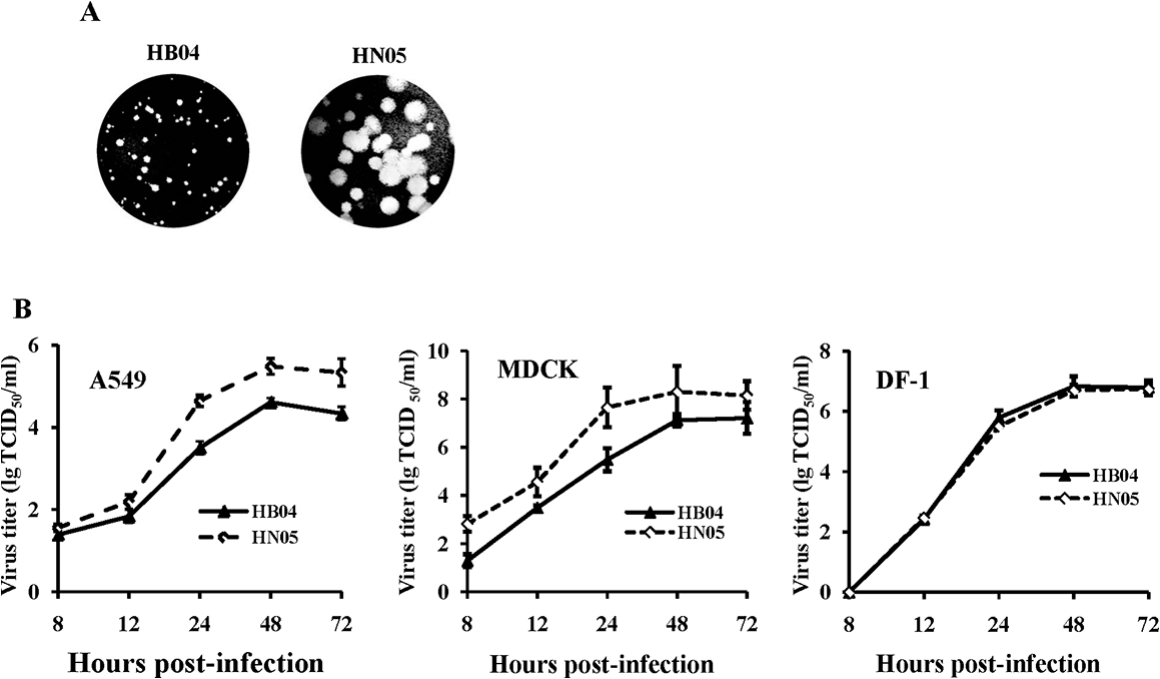
Growth characterization of two H5N1 isolates. (A) Plaque phenotypes of HB04 and HN05 viruses. Plaque assays were performed using MDCK cells under standard conditions followed by staining with crystal violet. (B) Replication kinetics of the HB04 and HN05 viruses *in vitro*. Monolayers of A549, MDCK, or DF-1 cells were infected at a multiplicity of 0.1, 0.01, or 0.01 PFU/cell with HB04 and HN05, respectively. Supernatants were collected at 8, 12, 24, 48 and 72 h p.i., and viral titers were determined using MDCK cells.

The avian influenza viruses A/chicken/Hubei/489/2004 (H5N1) (HB04) and A/duck/Hunan/316/2005 (H5N1) (HN05) are H5N1 isolates from chicken and wild ducks, respectively. Sequence analysis revealed that both isolates possess a polybasic amino acid stretch at the HA cleavage site (RERRRKK/R). There are several dozen different amino acids between the HB04 and HN05 isolates (Table 2); however, only a known mammalian-signature lysine at position 627 (627K) in the PB2 subunit [27,28,30,55] was observed in the HN05 virus. To characterize both H5N1 isolates *in vitro*, plaque formation and growth kinetics of HN05 and HB04 were measured in cells. In MDCK cells, HN05 formed large plaques with a mean size of 3.65 ± 0.61 mm, and HB04 had small plaques with a mean size of 0.70 ± 0.11 mm (Fig 1A). In A549 and MDCK cells, HN05 propagated more effectively than HB04. However, in the DF-1 chicken fibroblast cell line, HN05 and HB04 growth kinetics were identical, with peak titers of ~10^7^ TCID_50_/ml at 48 h p.i. (Fig 1B).

**Table 2:** Amino acid differences between the H5N1 influenza strains HB04 and HN05.

Genes	No. of amino acid differences	Residues with coding differences (HB04↔HN05)
PB2	10	S43P, I64M, A108T, V167I, N456S, M473V, V478I, **E627K**, T683K, T684A
PB1	5	K215R, R325K, T435I, L628Q, V644I
PA	22	D27N, L49S, S58G, I61M, T78I, G101D, T129I, F163L, Y176F, K204R, T259P, L261M, Y305C, G364S, S400P, A404S, V475A, S491T, V554I, G631S, G697D, A712T
HA	19	T52A, D59N, S100N, D110N, N140D, L154Q, K156T, S157P, S171N, V190I, P197S, R205K, E243D, L285V, M298I, R326K, Q338L, K345 (-), R513K
NP	5	S50R, K58N, I74V, S75R, D250N, V326I, S366N, G434S
NA	8	S50R, K58N, I74V, S75R, D250N, V326I, S366N, G434S
M1	0	—-
M2	2	S31N, A66E
NS1	9	L22F, A81T, R95G, D166G, I171N, F196Y, G204D, P210L, K224E
NS2	7	M14V, L19M, E36G, S44T, V52M, L58F, T105A

To test the pathogenicity of H5N1 viruses in animals, we first examined the virulence of HN05 and HB04 in chicken and mice. Based on OIE-defined criteria, both H5N1 isolates were highly pathogenic in chickens; HN05 and HB04 had intravenous pathogenicity indices (IVPI) of 1.4 and 2.4, respectively. In mice, HN05 replicated efficiently in the lungs, with an MLD_50_ of 10^2.4^ TCID_50_, but HB04 produced transient replication and an MLD_50_ greater than 10^5.4^ TCID_50_, suggesting that HB05 is highly pathogenic, and HB04 is nonpathogenic for mice.

### Generation of recombinant H5N1 viruses and their biological properties

To identify the genetic determinants of the high virulence of H5N1 viruses in mice, we used an eight-plasmid reverse genetics (RG) system to generate recombinant viruses [45]. Two eight-plasmid sets encoding individual genes of avian influenza viruses HN05 and HB04 were constructed to generate the RG HN05 and HB04 recombinant viruses by DNA transfection, respectively. Sequence analyses revealed that the genome sequences were identical in the RG and respective parental viruses.

**Fig 2:**
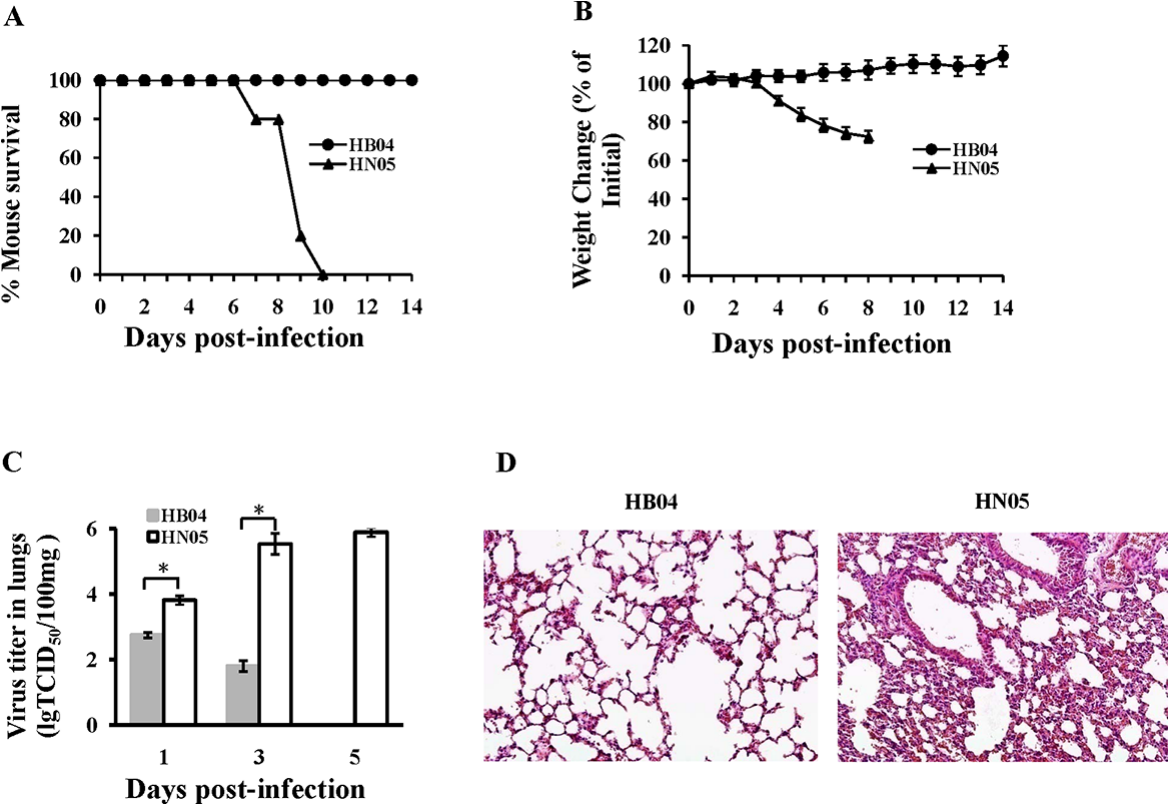
Pathogenicity of wt HB04 and HN05 viruses. (A) Survival rate of mice after intranasal inoculation with10^5^ TCID_50_ of the HB04 (n = 5) or HN05 (n = 5) virus. Mortality was monitored daily for 14 dpi. (B) Mean ± standard deviation (SD) of the percent body weight change of groups of mice (n = 5) after inoculation. (C) Mean viral titers ± SD in mouse-lung homogenates (n = 5) at 1, 3, and 5 dpi (*, *p<0*.*01*). (D) Representative histopathological changes in H&E-stained lung tissues from mice infected with HB04 or HN05 at 5 dpi (20×).

**Fig 3:**
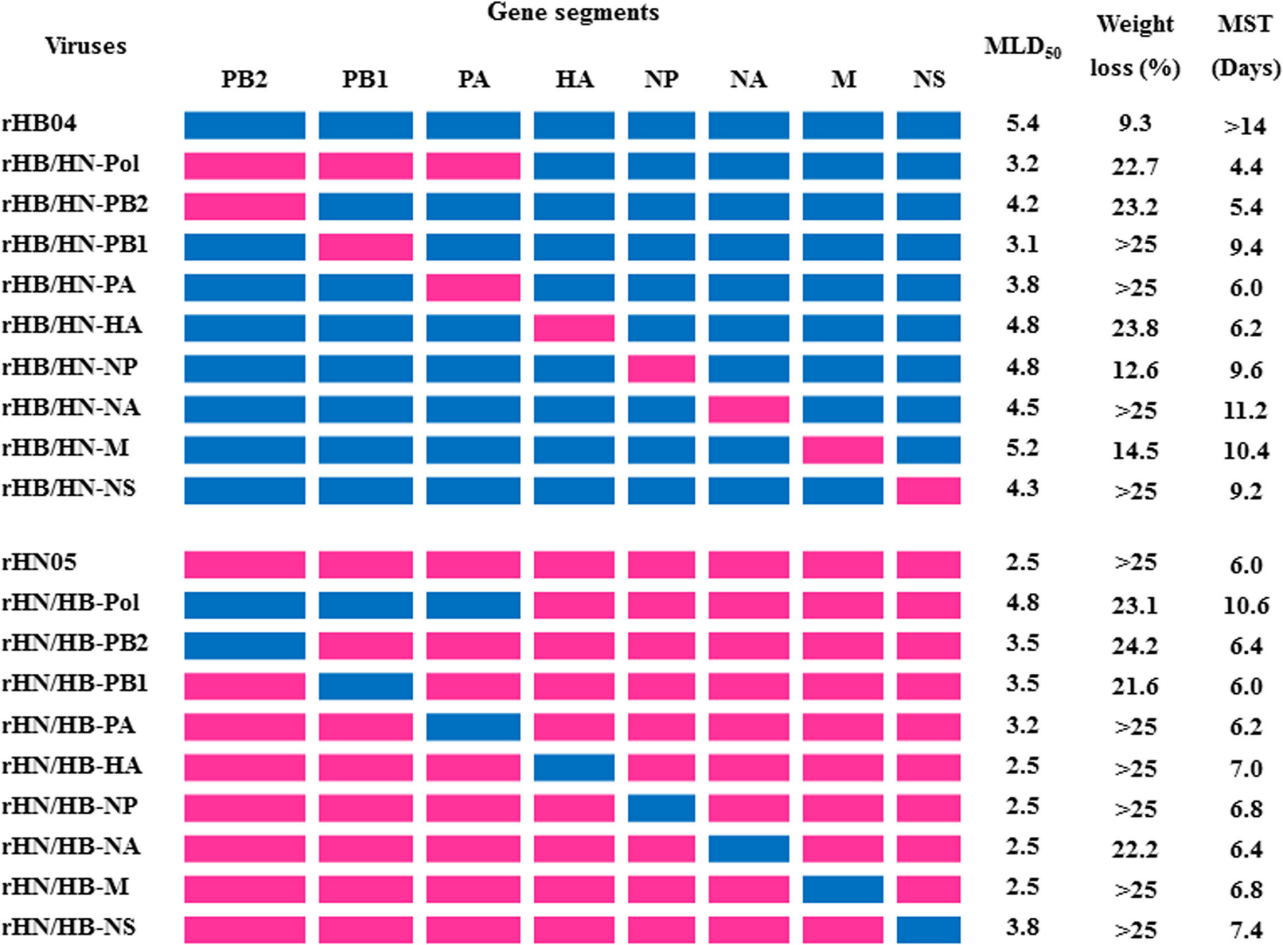
Pathogenicity of rescued reassortant viruses in mice. Colored bars indicate viral gene segments. Segments derived from HB04 and HN05 are shown in blue and red, respectively. The MLD_50_ of the rescued viruses was determined as described in the Materials and Methods section. The mean maximum weight loss was determined from five mice (percentage weight loss relative to day 0 p.i.) after infection with 10^5^ TCID_50_ of virus. The mean survival time (MST) of mice infected with 10^5^ TCID_50_ was calculated using the Kaplan-Meier method.

To test the pathogenicity of the RG viruses, we infected BALB/c mice with a dilution series of the recombinant viruses. Mice inoculated with 10^3^ TCID_50_ of HN05 exhibited clinical signs of disease, a hunched posture, ruffled fur, neurological symptoms, hind-limb paralysis, and considerable weight loss, and all these mice eventually died. By contrast, mice infected with the same dose of HB04 virus showed no sign of disease or body weight change. All mice infected with 10^5^ TCID_50_ of HB04 survived (Fig 2A and 2B). The rescued HN05 and HB04 viruses had MLD_50_ values of 10^2.5^ and 10^5.4^ TCID_50_, respectively (Fig 3), indicating that the rescued viruses maintained the same pathogenicity in mice as their parental viruses.

To examine the relationship between virulence and virus replication *in vivo*, we measured viral titers in the lungs of mice infected with HB04 and HN05 at 1, 3 and 5 dpi. HN05 replicated efficiently in the lungs of infected mice and attained a viral titer of ~10^6^ TCID_50_/100 mg at 5 dpi. In contrast, HB04 exhibited a low viral titer in the lungs at 1 and 3 dpi, and no virus was observed at 5 dpi (Fig 2C). Hematoxylin and eosin (H&E) staining analysis revealed that at 5 dpi, HN05-infected lungs showed extensive interstitial thickening and consolidation with inflammatory cell infiltrations in the alveolar and bronchial regions, whereas HB04-infected lungs displayed minimal signs of infection (Fig 2D). These results reveal that the high pathogenicity of HN05 in mice is positively related to enhanced replication and plaque formation in mammalian cells compared with the HB04 virus.

### Polymerase subunits are key determinants for the pathogenicity of H5N1 viruses in mice

To identify the genes that contribute to replication and virulence in mice, we generated eight single-gene recombinants, each containing one gene derived from HN05 and seven genes derived from HB04, and tested their pathogenicity in mice. The MLD_50_, maximum mean weight loss, and mean survival time (MST) were measured. Among these recombinants, viruses containing the M, HA, or NP gene derived from HN05 had little impact on the virulence of the parental HB04 (MLD_50_ at 10^5.2^–10^4.8^ TCID_50_), and the mean survival time (MST) following infection with 10^5^ TCID_50_ virus was 6.2–10.4 d; however, viruses containing the PB2, PB1 or PA gene of HN05 were significantly more virulent than HB04 (*p<0*.*01*). The MLD_50_ was determined to be between 10^4.2^ and 10^3.1^ TCID_50_, and the MST was 5.4–9.4 d (Fig 3). To further explore the potential synergistic effect of the polymerase subunits on virulence, the PB2, PB1 and PA genes of HB04 were simultaneously replaced with the corresponding HN05 genes to generate the rHB04/HN-Pol virus. The pathogenicity of the rHB/HN-Pol virus was similar to that of virus containing single PB1 gene replacement (Fig 3).

To examine the effect of individual HB04 genes on the virulence of rHN05, we generated eight single-gene recombinant viruses, each containing one gene from HB04 and seven from HN05. As expected, viruses containing the HB04-derived PB2, PB1 or PA gene were less lethal than the wt HN05 virus (MLD_50_, 10^3.2^ to 10^3.5^ *vs* 10^2.5^ TCID_50_), and the MST was 6–6.4 *vs* 6 d. Recombinants containing the HA, NP, NA or M gene of HB04 were as virulent as HN05. However, a recombinant virus containing all three HB04-derived polymerase genes was less virulent than any single polymerase gene recombinants (MLD_50_, 10^4.8^ *vs* 10^3.5^ to 10^3.2^ TCID_50_), and the MST was 6.4–7 *vs* 6 d (Fig 3).

### HN05 polymerase complex exhibits enhanced vRNP activity and viral replication *in vivo* and *in vitro*

**Fig 4:**
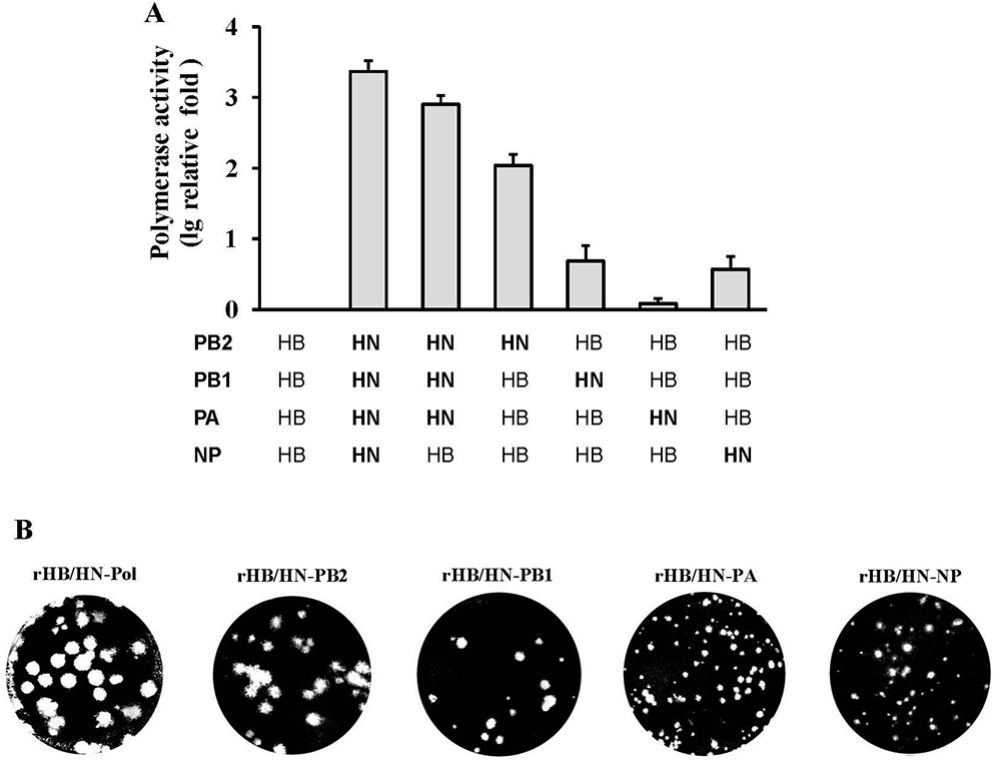
*In vitro* polymerase activities of vRNPs and plaque formation of recombinant viruses. (A) Polymerase activities of reconstituted HB04 and HN05 vRNP complexes composed of the indicated plasmids. 293T cells were transfected with the pPolI-NS-Luc plasmid and pRL-TK (internal control plasmid) as well as plasmids expressing PB2, PB1, PA and NP derived from either the HB04 or HN05 virus. Cells were incubated at 37°C for 24 h, and Firefly and Renilla luciferase activities were measured in the cell lysates. The data are represented as the means ± SD of the three independent experiments, expressed as log_10_ relative fold to HB04 RNP activity. (B) Plaque formation after virus titration in MDCK cells.

To study the effect of polymerase genes on viral ribonucleoprotein complex (vRNP) activity, we determined the polymerase activity of 7 vRNP combinations of PB2, PB1, PA and NP from HN05 or HB04 by measuring luciferase activity in a minigenome assay. We observed that the polymerase activity of HN05 vRNP was 10^3.4^-fold greater than that of HB04 vRNP, and rHN05 vRNP polymerase activity was reduced to 10^2.9^-fold of HB04 vRNP activity by replacement of NP with the HB04 NP segment. When the HB04 PB2 gene was replaced with the HN05 PB2 gene, there was a 10^2^-fold increase compared with that of HB04 vRNP in luciferase activity. Replacement of the PB1 or PA gene with the corresponding HN05 gene did not affect HB04 vRNP activity (approximately 1- to 5-fold increase in Luc activity). We next used MDCK cells to examine plaque formation by recombinants containing the PB2, PB1, or PA gene or all three polymerase subunit genes of HN05 in the HB04 backbone. We observed that rHB/HN-Pol formed larger plaques similar to HN05; rHB/HN-PB2 or rHB/HN-PB1 formed medium-sized plaques;, and rHB/HN-PA and rHB/HN-NP formed small plaques (Figs 1A and 4B).

**Fig 5:**
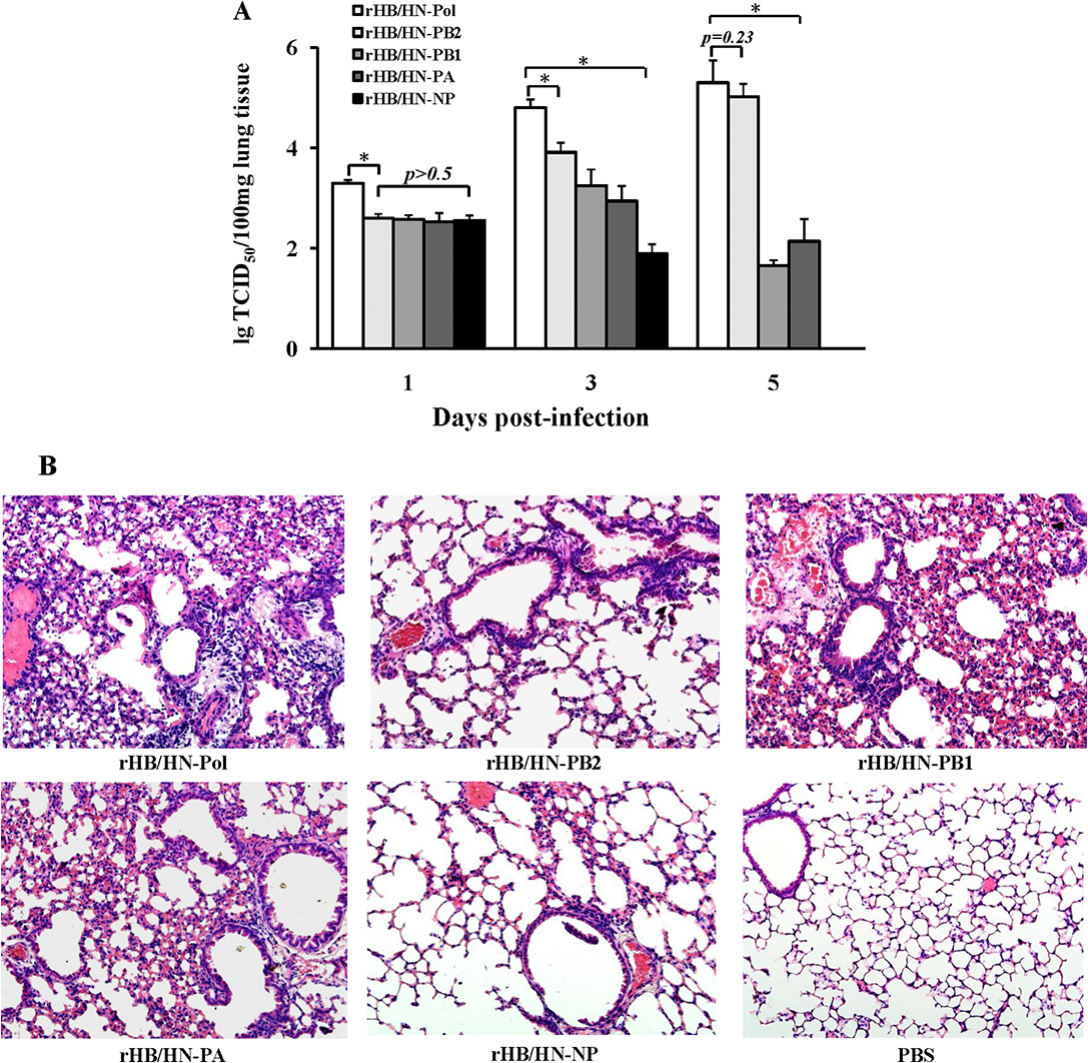
Replication *in vivo* and pathogenicity of recombinant viruses containing swapped polymerase genes in mice. (A) Six-week-old female BALB/c mice (n = 15) were infected intranasally with 2×10^3^ TCID_50_ of recombinant viruses. At the indicated time points, the infected mice (n = 5) were euthanized, and the viral titers in lungs were determined using MDCK cells (*, *p<0*.*01*). (B) Representative histopathological changes in H&E-stained lung tissues from mice infected with recombinant viruses at 5 dpi (20×).

To examine the replication of recombinants containing swapped polymerase genes *in vivo*, we measured viral titers in the lungs of mice infected with the reassortant virus rHB/HN-Pol, rHB/HN-PB2, rHB/HN-PB1, rHB/HN-PA, or rHB/HN-NP at 1, 3 and 5 dpi, respectively. We observed that the rHB/HN-Pol viral titers in the lungs were higher than any single-gene reassortant at 1 or 3 dpi (*p<0*.*01*). However, at 5 dpi, the rHB/HN-PB2 viral titer in the lungs of infected mice was similar to that of the rHB/HN-Pol virus; the rHB/HN-NP virus was not detected in the lungs of infected mice. The rHB/HN-PB1 and rHB/HN-PA viral titers were lower at 5 dpi than at 3 dpi (Fig 5A). To examine the relationship between pathogenicity and viral replication ability *in vivo*, the lung tissues of infected mice were analyzed by H&E staining at 5 dpi. Mice infected with rHB/HN-Pol had severe lung pathology including increased inflammatory infiltrates and hypertrophy of the alveolar lining cells, whereas mice infected with rHB/HN-NP had much less severe lung pathology. The severity of the lung pathology of mice infected with the single polymerase gene reassortants decreased in the following order: rHB/HN-PB1> rHB/HN-PB2> rHB/HN-PA. As expected, no histopathological change was observed in the PBS control mice (Fig 5B).

## Discussion

H5N1 avian influenza viruses are occasionally transmitted from poultry to humans and pose a grave threat to public health. In the case of the Hong Kong H5N1 outbreak in 1997, the molecular basis of increased virulence was studied in a mouse model [12]; however, the molecular determinants that enable H5N1 viruses to cross the species barrier from birds to mammals remain unclear. The viral polymerase is considered a determinant for host range, and changes within the polymerase complex, either by reassortment or mutation, facilitating the infection of a new species [13,32,46,56]. The selection on HA plays a main role in driving a bottleneck during the transmission of the reassortant H5N1viruses among mammals [16]. In the present study, we used a mouse model to examine the genetic basis of mammalian host specificity and virulence determinants in two previously uncharacterized H5N1 avian isolates. We observed that efficient viral replication *in vivo* is an important prerequisite for the pathogenicity of H5N1 viruses in mice. HN05 viral titers in the lungs of infected mice increased continuously after infection, and all the infected mice died; however, the HB04 virus did not replicate efficiently, and the viral infection in mice was completely cleared at 5 dpi. The replication efficiency of H5N1 isolates in mouse lungs was largely dependent on polymerase subunits, particularly PB2. These data are consistent with previous studies that PB2 is a determinant of host range in influenza viruses [27,32,46,57,58]. However, viral pathogenicity was not completely correlated with viral replication in mice. Among the recombinant viruses carrying a single polymerase gene, rHB/HN-PB2 replicated more efficiently and was less virulent in mice than rHB/HN-PB1 and rHB/HN-PA, which replicated less efficiently but were highly pathogenic.

To examine the effect of genetic background on virulence, we generated eight single-gene recombinant viruses, each containing seven genes from the parental HB04 background and one gene from the HN05 virus, or each containing seven genes from the parental HN05 background and one gene from the HB04 virus. We observed that, in addition to the NS gene, the H5N1 polymerase genes PB2, PB1 and PA were major virulence determinants and that the HA, NP, NA and M genes had a negligible effect on viral pathogenicity in mice in both the avirulent HB04 or virulent HN05 viral backgrounds. We calculated the MLD_50_ and MST in mice for the evaluation of both parental and recombinant viruses. Although the positive correlation between the MLD_50_ and MST values was observed in infected mice, there was not corresponding relationship between them. Similar results were also exhibited in previous studies [25,59,60]. Genome sequence analysis revealed the presence of a lysine at position 627 (627K) in the PB2 gene of the HN05 virus. The residue 627K of PB2 is considered a requirement for the high virulence of H5N1 and host range restriction in humans and mice [27,29,61]; however, some H5N1 viruses containing PB2 627E can also successfully infect mice and ferrets [8,14,30–32]. Although PB2 627E is considered an avian-signature residue, the pathogenicity or transmission of an H5N1 virus in ducks or chickens is not altered by PB2 627E or 627K [62], and the genetic stability of avian H5N1 viruses bearing PB2 627K depends on the viral lineage, and the genetic stability of H5N1 viruses with PB2 627K is also improved by other compensatory mutations [63]. Current studies demonstrated that H5N1 viruses containing three new high-pathogenicity-associated PB2-147T, -339T, and -588T amino acids in combination with PB2-627K display substantially higher pathogenicity than viruses with only the three new pathogenicity-associated amino acids or PB2-627K alone [61]. Additionally, the N66S amino acid mutation in PB1-F2 protein, which is encoded by the +1 open reading frame in the PB1 gene, has been shown to increase the pathogenesis of the of H5N1 virus [19,64]. In both HB04 and HN05 isolates, the predicted N66 amino acids were observed in the PB1-F2 protein and the three pathogenicity-associated amino acids in PB2 were not observed.

In A549 and MDCK cells, the HN05 virus had slightly faster replication kinetics than the HB04 virus. However, the growth kinetics of the HN05 virus was similar to that of HB04 in avian DF-1 cells. Minigenome assays revealed that the HN05 vRNP had 10^3.4^-fold greater polymerase activity than the HB04 vRNP. Among the recombinant vRNPs with one gene derived from HN05 in the HB04 background, the major contributor to polymerase activity was HN-PB2, followed by HN-PB1 and HN-PA. Notably, the recombinant vRNP containing HN-NP had 5-fold greater polymerase activity than wt HB04 vRNP, suggesting that NP regulated H5N1 replication to a certain extent *in vitro*. Previous studies have demonstrated that the H5N1 PB2 efficiently inhibits H1N1 RNP activity and viral replication by the interaction of the N-terminus of the PB2 subunit with the NP [65] and that the mouse-adapted PB2 gene of the influenza virus H3N2 conferred increased virulence and plaque size [46]. We monitored plaque formation by the parental H5N1 isolates and recombinants containing polymerase gene replacements; we observed that infection of MDCK cells with HN05 resulted in a large plaque size and that infection with HB04 resulted in smaller plaque sizes. The plaque-forming ability of recombinant viruses in MDCK cells decreased in the following order: rHB/HN-Pol > rHB/HN-PB2 > rHB/HN-PB1> rHB/HN-PA> rHB/HN-NP. The plaque-forming ability of recombinant viruses was consistent with the polymerase activity of vRNP complexes, but enhanced pathogenicity in mice did not correlate with the replication efficiency of recombinant viruses *in vitro* or *in vivo*, suggesting that the structure or properties of polymerase subunits might also contribute to H5N1 virulence in mice. Genome sequence analyses indicated that HN05 and HB04 differ at multiple amino acids, and the mammalian-signature-residue 627K was observed only in HN05 polymerase subunits, suggesting that host range restriction and pathogenicity are interrelated traits that involve multiple genes of viruses. The virulence markers for mammalian animals in HN05 polymerase genes were further examined. Although the BALB/c mouse was a useful model system for the evaluation of H5N1 virus pathogenesis [9,32,61], the pathogeneses of the HB04, HN05 and their recombinant viruses for other mammalian animals such as ferrets should be investigated to better understand interspecies transmissibility of the H5N1 viruses from their avian hosts to mammals.

In summary, we characterized two highly pathogenic H5N1 avian isolates and examined their pathogenicity in chickens and mice. We observed that the polymerase genes of the HN05 isolate are key determinants for viral replication ability *in vitro* and *in vivo*, as well as pathogenicity in mice. The PB2 subunit plays an important role in enhancing viral replication, and the PB1 and PA subunits contribute mainly to pathogenicity in mice. Our work provides additional evidence to understand the diversity and pathogenicity of H5N1 in different hosts.
